# Correction: Toward precision EEG: assessing the reliability of individual-level ERPs across EEG systems

**DOI:** 10.3389/fnhum.2026.1861549

**Published:** 2026-05-05

**Authors:** Frontiers Production Office

**Affiliations:** Frontiers Media SA, Lausanne, Switzerland

**Keywords:** auditory attention, event-related potentials, individual differences, mobile EEG, precision imaging, reliability

Author Adi Korisky's name was erroneously written as Adi Kilim.

The article citation was incorrectly written as: Kilim A, Rabinovitch E, Har-Shai Yahav P, McCandliss BD and Zion-Golumbic E (2026) Toward precision EEG: assessing the reliability of individual-level ERPs across EEG systems. *Front. Hum. Neurosci*. 20:1763477. doi: 10.3389/fnhum.2026.1763477. The correct citation is: Korisky A, Rabinovitch E, Har-Shai Yahav P, McCandliss BD and Zion-Golumbic E (2026) Toward precision EEG: assessing the reliability of individual-level ERPs across EEG systems. *Front. Hum. Neurosci*. 20:1763477. doi: 10.3389/fnhum.2026.1763477.

The copyright for this article was incorrectly written as: © 2026 Kilim, Rabinovitch, Har-Shai Yahav, McCandliss and Zion-Golumbic. This is an open-access article distributed under the terms of the Creative Commons Attribution License (CC BY). The use, distribution or reproduction in other forums is permitted, provided the original author(s) and the copyright owner(s) are credited and that the original publication in this journal is cited, in accordance with accepted academic practice. No use, distribution or reproduction is permitted which does not comply with these terms.

The correct statement is © 2026 Korisky, Rabinovitch, Har-Shai Yahav, McCandliss and Zion-Golumbic. This is an open-access article distributed under the terms of the Creative Commons Attribution License (CC BY). The use, distribution or reproduction in other forums is permitted, provided the original author(s) and the copyright owner(s) are credited and that the original publication in this journal is cited, in accordance with accepted academic practice. No use, distribution or reproduction is permitted which does not comply with these terms.

The original version of this article has been updated.

